# Adrenocortical Carcinoma: A Challenging Diagnosis

**DOI:** 10.7759/cureus.71998

**Published:** 2024-10-21

**Authors:** Marta Costa, Sónia Santos, Sofia Pereira, Daniel Aparício, Nelson Domingues

**Affiliations:** 1 Internal Medicine, Unidade Local de Saúde Viseu Dão-Lafões, Viseu, PRT

**Keywords:** adrenal cortex neoplasms, adrenocortical carcinoma, adrenocortical hyperfunction, endocrine gland neoplasms, neoplasm metastasis

## Abstract

Adrenocortical carcinoma (ACC) is a rare malignancy with aggressive behaviour and a poor prognosis. Patients can present with adrenal hormonal excess or with nonspecific symptoms driven by the presence of an abdominal mass or metastatic disease. Many are completely asymptomatic and diagnosed incidentally. ACC can cause considerable morbidity and mortality, mostly due to its ability to invade surrounding tissues, produce hormones, and spread to distant organs. The authors describe a case of a 62-year-old woman who presented with subacute dorso-lumbar pain. A computed tomography (CT) scan revealed osteolytic lesions in her dorsal spine and sacrum, suggesting metastatic disease. Later on, she presented with hypercortisolism and refractory hypokalemia, so an abdominal and pelvis CT was performed, which showed a suspicious mass in the right adrenocortical gland. A CT-guided adrenal biopsy confirmed ACC. Unfortunately, our patient's clinical status rapidly deteriorated, resulting in her death only a few weeks later. ACC is often found at an advanced stage and with distant metastases, most commonly in the liver, lungs, lymph nodes, and bone. The overall prognosis of ACC is generally poor, but it varies depending on the extent of the disease. Multiple factors have been shown to be relevant in the prognostic classification, such as tumor stage, cell proliferation markers, and resection status. Currently, the only curative treatment is complete surgical resection. Adjuvant therapies have often been shown to decrease recurrence rates or as an alternative in patients with advanced disease. Many studies have been conducted to better understand the molecular basis of ACC, thus enabling the classification into molecular subtypes, but more studies are necessary to identify targets amenable to pharmaceutical intervention. With this case report, we want to emphasize that the diagnosis of ACC is not always obvious. Although metastases are infrequent, their presence is by far the strongest indicator of poor prognosis. All patients with proven or suspected ACC benefit from multidisciplinary monitoring, preferably at a specialized center.

## Introduction

Adrenocortical carcinoma (ACC) is a rare malignancy with aggressive behaviour and a poor prognosis [1,2]. Even though ACC is the most-common primary cancer in the adrenal gland [3] and the second-most common malignant tumor of an endocrine organ [4], it has an incidence rate of 0.7-2.0 per million population per year [5], meeting the criteria of “orphan” disease in the European Union and the United States [3]. ACC is more frequent in women, with a female-to-male ratio between 2.5 and 3 to 1 [6]. It develops mostly between the fifth and seventh decades of life [7], with a smaller peak in children under five years old [6].

Most cases develop sporadically and are often discovered as incidentalomas. In the past few decades, research has found that ACC may be driven by a myriad of genetic and epigenetic aberrations [3]. Less than 10% are associated with genetic syndromes (for example, multiple endocrine neoplasia type 1, Li-Fraumeni, Lynch, Beckwith-Wiedemann syndromes [1,7], Neurofibromatosis type 1, Carney Complex, and Werner syndrome) [8]. Other etiologic risk factors have been suggested, such as smoking and increased estrogen exposure from contraceptive use or during pregnancy [8].

Patients can exhibit adrenal hormonal excess, with either hypercortisolism presenting with Cushing syndrome features or hyperandrogenism resulting in male pattern baldness, virilization, hirsutism, menstrual abnormalities, or both. It can also display nonspecific symptoms driven by the presence of an abdominal mass (such as local tumor growth, abdominal or flank pain, etc.) or metastatic disease (for example, jaundice and bone pain ) [6]. Many are completely asymptomatic and diagnosed incidentally [6] since classic malignancy-associated symptoms are rarely present, and clinical signs can be masked due to their fast progression [9].

A variety of questions and controversies still exist regarding diagnosis and treatment, as only a small number of trials have been performed due to the rarity of this cancer [3]. ACC can cause considerable morbidity and mortality, mostly due to its ability to invade surrounding tissues, produce hormones, and spread to distant organs [5]. The present case has already been presented at the 19^th^ European Congress of Internal Medicine in March of 2021.

## Case presentation

**Figure 1:**
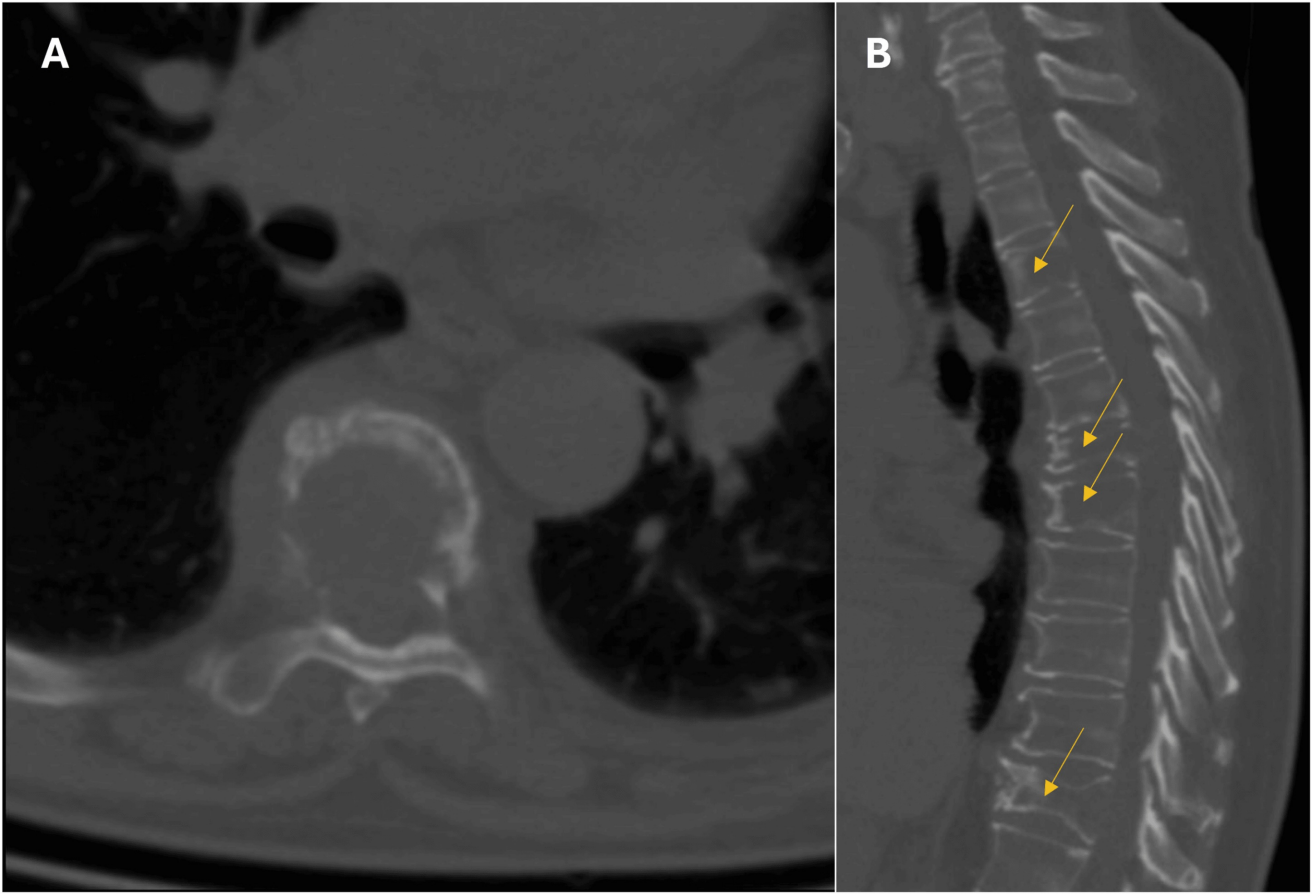
Dorsal spine CT Axial view (A) showing an extensive area of osteolytic process with severe involvement of the vertebral body and the right posterior arch of the sixth dorsal vertebra. Coronal view (B) highlighting multiple areas of radiolucency in the third, sixth, seventh and eleventh dorsal vertebrae suggesting metastatic lesions (yellow arrows).

We describe the case of a 62-year-old woman who presented with subacute moderate-to-intense dorso-lumbar pain. She described a localized pain with a mechanical pattern radiating to the left knee, to which painkillers (acetaminophen, tramadol, and non-steroidal anti-inflammatory drugs) only provided partial relief. Additionally, she mentioned paresthesia in both legs and movement impairment, with a functional impact on her daily activities. Computed tomography (CT) scan revealed osteolytic lesions in her dorsal spine and sacrum, suggesting metastatic disease (Figure 1). She was later admitted for further studies and submitted to a bone biopsy that initially suggested Multiple Myeloma.

**Figure 2:**
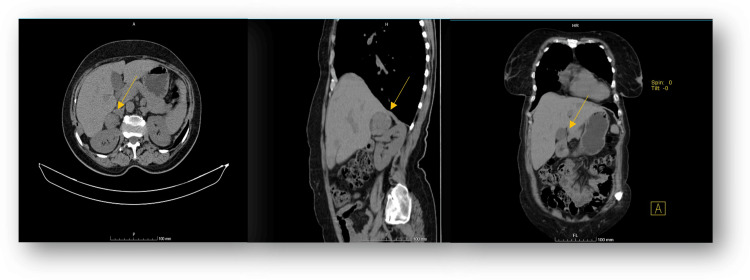
Chest, abdomen and pelvis CT CT scan images (axial, sagittal and coronal view - from left to right) showing a 5x4.4 cm rounded, expansive formation in the right adrenocortical gland (yellow arrows) revealing punctiform parietal calcifications.

She later underwent bone radiotherapy. However, she then developed hypercortisolism and refractory hypokalemia, which was inconsistent with the initial diagnosis of multiple myeloma. A histological revision was made, broadening the differential diagnosis to ACC, melanoma, and PEComa (Perivascular Epithelioid Cell Tumor). Our patient underwent careful clinical assessment, hormonal workup, and imaging. A chest, abdomen, and pelvis CT was performed showing a suspicious mass in the right adrenocortical gland (Figure 2).

Hormonal workup searching for glucocorticoid excess, sex steroids, steroid precursors, and mineralocorticoid excess revealed high 24-hour (24h) urine cortisol and hypokalemia, without other disorders. A pheochromocytoma was excluded by normal fractionated metanephrines in 24h urine measurement (Table 1). Head MRI also excluded Cushing’s Disease and brain metastases.

**Table 1:** Biochemical and hormonal workup ACTH - Adrenocorticotropic hormone; DHEA-S - Dehydroepiandrosterone sulfate * Androstenedione, 17-beta-estradiol and 11-deoxycortisol were unavailable at any local laboratory.

	Results	Reference values
Glucocorticoid excess		
Basal ACTH (plasma)	2.2	4.7-48.8 pg/mL
Urinary cortisol	804	28-214 ug/24h
Sex steroids and steroid precursors*		
DHEA-S	200	32-204 ug/dL
17-hydroxyprogesterone	1.3	0.13-0.51 ng/mL
Testosterone	18.48	< 7-48.93 ng/dL
Mineralocorticoid excess		
Serum potassium	2.2	3.5-5 mEq/L
Serum aldosterone	48.7	41-323 pg/mL
Serum renin	16.7	4.4-46.1 uUI/mL
Catecholamine excess		
24-h urinary fractionated metanephrines		
Total metanephrines	440	329-1263 ug/24h
Metanephrine	56	64-302 ug/24h
Normetanephrine	187	162-527 ug/24h
3-methoxytyramine	197	103-434 ug/24h
Plasma catecholamine		
Adrenalin	18	< 84 ng/L
Noradrenalin	276	< 420 ng/L

Finally, a CT-guided adrenal biopsy confirmed ACC, and she was promptly referred to a specialized center. Unfortunately, our patient's clinical status rapidly deteriorated, compromising her general health and making further invasive therapeutic procedures unadvisable. She received palliative care and comfort measures, focusing on treating end-of-life syndrome symptoms such as nausea, pain, and dyspnea, as well as anxiety and delirium, until her death a few weeks later. Her close relatives were involved and also received psychological and spiritual support.

## Discussion

ACC is often found at an advanced stage and with distant metastases [7], most commonly in the liver, lungs, lymph nodes, and bone [3]. Unusual metastatic locations include the stomach, pancreas, skin, spleen, tongue, and brain, including meninges [4]. Despite the lack of evidence, experts advise performing adrenal-focused imaging, biochemical and hormonal workups, and preferably confirmation by histological results [9]. Biopsy of an adrenal lesion is not usually recommended [10], although it may be considered in patients with metastatic ACC, especially in cases when surgery is not an option, and further information could help guide oncological management [3]. It is also important to perform additional imaging if metastatic lesions are suspected [9].

The overall prognosis of ACC is generally poor but varies depending on the extent of the disease [3]. Multiple factors have been shown to be relevant in prognostic classification, such as tumor stage, cell proliferation markers (mostly proliferation index Ki67 in this particular case), and resection status [11]. Hormonally functional status is also an important predictor of poor prognosis, as it seems to be in all types of hormonally-active tumors, most likely due to their systemic and immunosuppressive impact [2]. Other parameters include age, genetic profile, surgical approach, and the use of adjuvant therapy [2]. 

Currently, the only curative treatment is complete surgical resection [3]. Adjuvant therapies are often added to decrease recurrence rates or as an alternative in patients with advanced disease [11]. An additional challenge in treatment is to mitigate the harmful effects of excess hormone secretion, which is a great cause of morbidity and impairment in quality of life [3]. We would like to highlight the role of mitotane as the only chemotherapy agent proven to increase overall and recurrence-free survival [12]. Moreover, local therapies, such as radiotherapy, chemoembolisation, radioembolisation and image-guided thermal ablation, should also be considered to prevent metastatic complications, mass effects, and neurologic symptoms [11].

More recently, immunotherapy has emerged as a new pillar for many solid tumors, and it is a promising treatment for ACC [3]. The role of chronic inflammation is also a topic of interest, as there is a known association between several inflammatory biomarkers and survival rates in some cancers. Therefore, an inflammation-based score may be included in prognostic stratification [13]. Many studies have been conducted to better understand the molecular basis of ACC, thus enabling the classification into molecular subtypes. There is still no specific correlation between specific mutations and potential response to treatment [12], resulting in no clear improvement in survival rates [10]. More studies are necessary to identify targets amenable to pharmaceutical intervention [3].

## Conclusions

With this case report, we want to emphasize that the diagnosis of ACC is not always obvious. Although metastases are infrequent, their presence is by far the strongest indicator of poor prognosis. Metastatic ACC in the spine is exceedingly rare and has no standard curative management yet, although mitotane and local therapeutic measures are currently the treatment of choice in advanced ACC and are not amenable to complete surgical resection.

ACC is often incurable and the burden is often greater in these patients. All patients with proven or suspected ACC benefit from a multidisciplinary monitoring, preferably at a specialized center. This multidisciplinary team may include endocrinologists, surgeons, oncologists, pathologists and geneticists, as well as nursing and palliative care for patients and their families.
